# The Associations between Two Vital GSTs Genetic Polymorphisms and Lung Cancer Risk in the Chinese Population: Evidence from 71 Studies

**DOI:** 10.1371/journal.pone.0102372

**Published:** 2014-07-18

**Authors:** Kui Liu, Xialu Lin, Qi Zhou, Ting Ma, Liyuan Han, Guochuan Mao, Jian Chen, Xia Yue, Huiqin Wang, Lu Zhang, Guixiu Jin, Jianmin Jiang, Jinshun Zhao, Baobo Zou

**Affiliations:** 1 Department of Preventative Medicine, Zhejiang Provincial Key Laboratory of Pathological and Physiological Technology, School of Medicine, Ningbo University, Ningbo, Zhejiang Province, People's Republic of China; 2 Department of Science Research and Information Management,Zhejiang Provincial Center for Disease Control and Prevention, Hangzhou, Zhejiang Province, People's Republic of China; 3 Municipal Center for Disease Prevention and Control of Ningbo City, Ningbo, Zhejiang Province, People's Republic of China; 4 Department of Epidemiology and Health Statistic, Anhui Medical University, Hefei, Anhui Province, People's Republic of China; 5 School of Health Management, Anhui Medical University, Hefei, Anhui Province, People's Republic of China; MD Anderson Cancer Center, United States of America

## Abstract

**Background:**

The genetic polymorphisms of glutathione S-transferase (GSTs) have been suspected to be related to the development of lung cancer while the current results are conflicting, especially in the Chinese population.

**Methods:**

Data on genetic polymorphisms of glutathione S-transferase Mu 1 (*GSTM1*) from 68 studies, glutathione S-transferase theta 1 (*GSTT1*) from 17 studies and *GSTM1-GSTT1* from 8 studies in the Chinese population were reanalyzed on their association with lung cancer risk. Odds ratios (OR) were pooled using forest plots. 9 subgroups were all or partly performed in the subgroup analyses. The Galbraith plot was used to identify the heterogeneous records. Potential publication biases were detected by Begg's and Egger's tests.

**Results:**

71 eligible studies were identified after screening of 1608 articles. The increased association between two vital GSTs genetic polymorphisms and lung cancer risk was detected by random-effects model based on a comparable heterogeneity. Subgroup analysis showed a significant relationship between squamous carcinoma (SC), adenocarcinoma (AC) or small cell lung carcinoma (SCLC) and *GSTM1* null genotype, as well as SC or AC and *GSTT1* null genotype. Additionally, smokers with *GSTM1* null genotype had a higher lung cancer risk than non-smokers. Our cumulative meta-analysis demonstrated a stable and reliable result of the relationship between *GSTM1* null genotype and lung cancer risk. After the possible heterogeneous articles were omitted, the adjusted risk of GSTs and lung cancer susceptibility increased (fixed-effects model: OR_GSTM1_ = 1.23, 95% CI: 1.19 to 1.27, P<0.001; OR_GSTT1_ = 1.18, 95% CI: 1.10 to 1.26, P<0.001; OR_GSTM1-GSTT1_ = 1.33, 95% CI: 1.10 to 1.61, P = 0.004).

**Conclusions:**

An increased risk of lung cancer with *GSTM1* and *GSTT1* null genotype, especially with dual null genotype, was found in the Chinese population. In addition, special histopathological classification of lung cancers and a wide range of gene-environment and gene-gene interaction analysis should be taken into consideration in future studies.

## Introduction

Lung cancer is the most common malignancy in the world and the leading cancer in males, accounting for 17% of the total new cancer cases and 23% of the total cancer deaths [1]–[3]. The burden of lung cancer mortality in females in developing countries is up to 11% of the total female cancer deaths [2]. In the United States, there were 226,160 newly diagnosed cases and 160,340 deaths due to lung cancer in 2012 [4]. In China, although females have a lower prevalence of smoking, there is still higher lung cancer rates (21.3 cases per 100,000 females) than those in European countries [5], due to indoor air pollution, cooking fumes, occupational and environmental pollutions. Besides, due to the incurable nature and less than a five-year survival rate (only 16%), lung cancer has attracted a huge attention across the whole world [6].

Lung cancer can be divided into several types by pathological classification, such as squamous cell carcinoma (SC), adenocarcinoma (AC) and large or small cell carcinoma. It is also classified as small cell lung carcinoma (SCLC) and non-small cell lung carcinoma (NSCLC), which accounts for about 85% of all lung cancer [7]. Given the possible relapses in the local respiratory system and the metastasis in other systems after the classical treatments of radical surgery, immunotherapy has provided an innovative method for lung cancer treatment in the past 30 years to enhance the clinical outcome, alleviate the disease burden, prevent recurrences and attenuate toxicity [8]–[14].

Tobacco smoking has clearly been demonstrated to be a strong exogenous factor for lung cancer risk [15]–[17]. Polycyclic aromatic hydro-carbons (PAHs) and the tobacco-specific nitrosamine 4-(methylnitrosamino)-1-(3-pyridyl)-1-butanone (NNK) are considered to be the major carcinogens, which can interact with DNA and cause the formation of DNA adducts [17]. In the meantime, free radicals from tobacco smoking can induce oxidative damage to lung tissues, and also damage DNA, which provides another clue to lung cancer development [18]–[21]. In this process, DNA was damaged by superoxide anions (O_2_^−^) and hydroxyl radicals (OH^−^) and was repaired by antioxidant enzymes. This balance can be broken by both environmental and genetic factors. Available molecular epidemiology studies have shown that genetic polymorphisms play a major role in the progress of carcinoma [22], [23]. Among these studies, genetic variants of carcinogen-metabolizing enzymes have received much attention, especially glutathione S-transferase (GST) genes and cytochrome P450 genes. The cytochrome P450 (CYP450) family, as the first-pass metabolism enzymes, plays an important role in many physiological and biochemical reactions in the human body, and participates in the metabolic process of endogenous and exogenous substrates (biosynthesis and degradation) [24]. Toxic materials like benzo[a]-pyrene and other PAHs could be metabolized to oxygenated intermediates and then degraded sequentially to lower toxic or non-toxic substances by the second-pass metabolic enzymes such as the glutathione S-transferases (GSTs) family [25], [26]. Therefore, the polymorphisms of both gene families might affect the metabolism of tobacco toxicants in lung and finally influence the advancement of cancer.

The GSTs family can detoxify environmental carcinogens and toxins, oxidative stress products, and several covalent conjugated electrophilic compounds [27], [28]. *GSTM1* and *GSTT1* are two critical GSTs family genes, separately encoded mu and theta GST classes and located in 1p13.3 and 22q11.23 in the human chromosome, respectively. The common *GSTM1* polymorphisms include three alleles, GSTM1*A, GSTM1*B and GSTM1*0, where GSTM1*0 means a null mutation [29]. Another gene, *GSTT1* is polymorphic with two alleles (GSTT1*1 and GSTT1*0). The homozygous combinations of GSTM1*0 allele as a null genotype could lead to a functional deficiency [29], as well as GSTT1*0 [30], while other genotypes remain functional [31]–[34].

Most molecular epidemiologic studies suggested an association between GST genetic polymorphisms and lung cancer risk, especially when deletion of *GSTM1* is observed in the Asian population [35]–[44]. However, the current research results are conflicting, especially in the Chinese population [36], [38], [42], [44]–[46]. Due to the difference in sample size, smoking status and environmental factors, etc., conflicting or vague results were found in these studies.

To identify the association of two vital GST genetic polymorphisms (*GSTM1* and *GSTT1*) with lung cancer risk, an updated systematic meta-analysis was performed in this study by selecting all eligible studies in the Chinese population.

## Methods

### 1. Literature research strategy

A computer-based literature search was carried out in EMBASE, PubMed, ISI Web of Knowledge, Chinese Biomedical Database (CBM), VIP database, Chinese National Knowledge Infrastructure (CNKI), and Wanfang Data (the latest research retrospect until October 2013) to collect articles related to the association of *GSTM1* and/or *GSTT1* polymorphisms and lung cancer susceptibility in the Chinese population. Additionally, relevant references of the articles were also collected. We also searched two websites (http://www.baidu.com and http://scholar.google.com) to identify additional eligible studies. MeSH terms (“glutathione S-transferase” or “GST” or “GSTM1” or “GSTT1”) and (“lung carcinoma” or “lung cancer” or “lung neoplasms”) and (“China” or “Chinese” or “Taiwan”) were used in the databases. Eligible research articles not captured by the above research strategies were further searched by bibliographies without language limitation.

### 2. Inclusion and exclusion criteria

Inclusion criteria: (1) individuals or samples in all eligible studies were examined and diagnosed by polymerase chain reaction (PCR), pathologic diagnosis or other methods to get a full picture of GST genetic polymorphisms and lung cancer types; (2) Chinese living in China; (3) articles providing raw data including odds ratio (OR) with 95% confidence interval (CI) and respective variance, or the relevant information could be calculated.

Exclusion criteria: (1) Chinese out of China; (2) raw data not available; (3) when there were multiple publications by the same researchers, only the latest or the largest population study was adopted; (4) meeting abstract, case reports, editorials, newsletter and review articles were excluded.

### 3. Data extraction and synthesis

To decide inclusively or exclusively, articles were identified by three independent work groups (group 1-Kui Liu and Lu Zhang; group 2-Xia Yue and Xialu Lin; group 3-Jian Chen and Guixiu Jin) using a standardized data extraction form designed by ourselves. Discrepancies among three groups were further discussed by all parties. If consenses was still not reached, another group (group 4-Huiqin Wang and Qi Zhou) would make the final decision. Firstly, the titles and abstracts of all studied articles were screened to determine their relevance. If the titles and abstracts were ambiguous, full articles would be investigated. In order to make full use of the available data, it was counted as two separated studies if two different control groups were employed in the same article, such as two different controls versus the same control. If there were more than one region to be investigated in one article, information for each region was also counted as a separated study. Information collected from each eligible study included: first author, year of publication, region, study time, pathologic diagnosis, source of control, characteristics of cases and controls, genotype frequency of null *GSTM1*, null *GSTT1*, and null of both genotypes (Table 1). Hardy Weinberg Equilibrium(HWE) argues that genotype frequencies at any locus are a simple function of allele frequencies under the precondition of no migration, mutation, natural selection, and assortative mating [47]. HWE test was usually assessed in the control group [48]. Furthermore, details of eligible studies used for detecting GSTs genotype, combined evaluation of other genes, HWE test results of *CYP1A1* polymorphisms, the percent of null GSTs genotype in the control groups, smoking status, study type and quality score were also elicited (Table 2). Study types also consisted of epidemiological design and non-epidemiological design. Epidemiological designs were comprised of case-control, cohort and nested case-control studies, all of which must satisfy three conditions for both cases and controls: explicit diagnosis of status (histology or cytology), clear description of the age period, and the same source population [49]. Those not meeting the conditions were considered non-epidemiological designs. The quality score of epidemiological studies was evaluated by Newcastle-Ottawa Scale (NOS).

**Table 1:** Characteristics of the studies related to the effects of GSTs genetic polymorphisms and lung cancer risk.

No.	First author(ref.)	Region	Study time	Pathologic diagnosis^¶^	Sourceof controls	Characteristic of Cases	Characteristic of Controls	Null *GSTM1*/Group number		Null *GSTT1*/Group number		Dual Null/Group number	
								case	control	case	control	case	control
1*	Liu DZ 2012 [54]	Heilongjiang (Harbin)	2010–2012	ALL	Population	360 cases in Han population (142 SC, 140 AC, 37 SCLC, 41 others)	360 cancer-free controls matched by gender and age in Han population	145/360	107/360				
2	Wang N 2012 [55]	Henan	2008.2–2008.8	ALL	Population	209cases(103 SC, 69 AC, 28 SCLC and 9 others	256 controls, comparable in age and gender in Han population	122/209	113/256	90/209	100/256		
3*	Li WY 2012 [56]	Beijing	2005.8–2006.6	ALL	Population	217 cases (NSCLC)	198 healthy controls with comparable in age and gender	127/217	93/198				
4	Chen CM 2012 [57]	Zhejiang	NA	ALL	Population	200 cases (59 AC, 104SC, 37 other NSCLC)	200 controls without any tumor with comparable in gender and age	123/200	110/199				
5	Yao ZG 2012 [58]	Beijing	2006.6–2010.6	ALL	Population	150 cases including 97 males and 53 females	150 healthy controls including 89 males and 61 females	96/150	68/150				
6	Liu JN 2012 [59]	NA	NA	NA	Population	100 cases including 29 SC, 40 AC, 18SCLC and 13 mixed style	135 healthy controls with comparable in gender, age and smoking status in Han population			57/100	56/135		
7	Han RL 2012 [60]	InnerMongolia	NA	ALL	Hospital	128 cases	214 hospital controls without tumors, rheumaticdisease and pulmonary disease	79/128	89/214				
8*	Jin YT 2011[61]	Anhui	2006–2007	ALL	Hospital	154 cases (NSCLC)	154 controls without any tumors and chronic respiratory disease, matched by age, gender and ethnicity.	64/154	58/154				
9	Ai C 2011 [62]	NA	2007.5–2010.5	ALL	Population	50 cases (38 males)	50 controls with comparable in gender, age, ethnicity, smoking status and occupational group	36/50	23/50				
10	Zhang JQ 2011 [63]	Yunnan (Xuanwei)	NA	ALL	Population	50 cases	50 controls, comparable in gender, age, residential township, weight and combustion method of coal	34/50	22/50				
11	Du GB 2011 [64]	Sichuan	NA	ALL	Hospital	125 cases (57 SC, 31 AC, 37 others)	125 controls with comparable in age and gender	73/125	71/125				
12	Li Y2011 [65]	Henan (Zhengzhou)	2003–2006	ALL	Population	103 cases including 64 SC, 13 AC,21SCLC and 5 others	138 healthy controls, comparable in age and gender	63/103	61/138				
13	Bai TY 2011[66]	InnerMongolia	2006–2009	ALL	Hospital	106 cases	250 controls without tumors, rheumaticdisease and pulmonary disease			50/106	111/250		
14*	Jin YT 2010[67]	Anhui	2005.6–2007.12	ALL	Hospital	150 cases (83 SC, 33AC, 34 mixed types)	150 controls matched by age and gender.	95/150	79/150				
15	Zheng DJ 2010[68]	Tianjin	2008.3–2009.7	ALL	Population	265 cases including 120 SC, 99AC, 23 SCLC and 23 others	307 healthy controls without respiratory disease and family history of lung cancer, comparable in age and gender	150/265	175/307				
16	Zhu XX 2010[69]	Hunan	2009.3–2009.12	ALL	Population	160 female cases (19SC, 109AC, 17SCLC, 15 others)	160 healthy female controls, comparable in age and residential township	93/160	72/160				
17	Fan J 2010 [70]	Guangxi	2009.3–2010.5	ALL	Population	58 cases	60 healthy controls, comparable in age and residential township	40/58	33/60	38/58	29/60	29/58	20/60
18	Chang FH 2009 [71]	InnerMongolia	NA	NA	Population	263 cases	263 healthy controls matched by age, gender and ethnicity	152/263	126/263				
19	Chen H 2008 [72]	Anhui	2005.9–2007.12	ALL	Population	158 cases (86 SC, 36AC, 36 other)	455 controls with comparable in gender and age	99/158	246/454				
20	Liu Q 2008[73]	Shandong	2006.3–2007.5	PARTIAL	Population	110 cases (70 males) including 68 SC and 1 AC, 11 others	125 controls (82 males) matched by age and gender	66/110	57/125				
21	Qi XS 2008 [74]	Gansu	2005–2007	ALL	Hospital	53 cases (27SC, 3 AC, 230 others)	72 controls with comparable in gender and smoking status	Δ34/53	Δ41/72	17/53	27/72	10/53	17/72
22	Xia Y 2008 [75]	Gansu (Qinyang)	2005–2007	ALL	Hospital	58 cases (age in 40–75 years, 52 males)	116 controls (age in 38–75 years, 104 males)	34/58	61/116				
23	Gu YF 2007[76]	Beijing	2000.11–2005.6	ALL	Hospital and Population	279 cases (84 SC, 110 AC, 45 SCLC and others 40)	684 (575 healthy controls and 109 benign pulmonary disease cases) equally with comparable in age, gender and ethnicity	164/279	325/684				
24	Wang YS 2007 [77]	Anhui	NA	ALL	Population	47 NSCLC (31 SC, 7 AC, 9 others)	94 healthy controls (84 males) with comparable in age and gender	27/47	50/94				
25	Lei FM 2007 [78]	Sichuan (Chengdu)	2004.1–2006.1	NA	Population	42 cases (age 64.7±11.03 years)	103 controls (age 50.8±7.02 years) with comparable in residential township, gender and occupation	24/42	57/103				
26	Chang FH 2006 [79]	InnerMongolia	NA	ALL	Hospital	163 cases (92 males)	163 controls without tumors, rheumaticdisease and pulmonary disease, matched by age, gender, residential township	106/163	78/163				
27*	Chen HC 2006 [80]	Hunan	NA	ALL	Population	97non-smoker cases (42 males) including 51 SC, 43 AC, 3 unknown)	197 healthy controls (96 males) matched by age and gender in non-smokers	60/97	89/197	59/97	85/197	36/97	44/197
28	Li Y 2006 [81]	Henan	2003.3–2003.8	ALL	Population	98 cases including 64 SC, 13 AC and 21 SCLC	136 controls, comparable in age and gender	60/98	60/136				
29	Yao W 2006 [82]	Henan (Zhengzhou)	NA	ALL	Population	77 cases including 42 SC, 24 AC and 11 others	107 healthy controls (57 males)	45/77	45/107	44/77	54/107	26/77	25/107
30	Qian BY 2006 [83]	Tianjin	2004.3–2005.3	ALL	Population	108 cases in han population in Tianjin city	108 controls (66 males) with comparable in age and occupational status	69/108	53/108				
31	Wang QM 2006 [84]	NA	NA	PARTIAL	Population	56 cases (age 64.86±12.53 years, 50 males)	42 controls (age 59.12±12.51 years, 38 males)	40/56	19/42				
32	He DX 2006[85]	Yunnan (Kunming)	NA	NA	Population	61 cases (age in 40–60 years)	46 healthy controls (age in 40–55 years)			33/61	29/46		
33*	Chan EC 2005 [86]	NA	NA	ALL	Population	75 cases (31 SC and 44 AC)	162 healthy controls without history of pulmonary disease, matched by age and gender	31/75	91/162				
34	Yuan TZ 2005 [87]	Sichuan	NA	ALL	Population	150 cases (70 SC, 61 AC, other 19)	152 controls with comparable in age and gender in Han population			82/150	58/152		
35	Li DR 2005 [88]	Sichuan	2001.7–2004.2	ALL	Hospital	99 NSCLC cases (age 58.4±10.6 years,74 males) including 41 SC, 42 AC, 16 mixed style	66 controls (age 42.4±14.9 years, 37 males) with lung benign disease.	57/99	27/66				
36	Ye WY 2005 [89]	Guangdong (Guangzhou)	NA	ALL	Hospital	58 cases	62 controls without tumor and respiratory disease, comparable in age and gender	23/58	33/62				
37*	Chou YC 2005 [90]	Taiwan	1990.7–2000.12	NA	Population	30 cases	60 cancer-free controls matched for gender, age and residential township	18/30	39/60				
38	Liang GY 2004[91]	Jiangsu (Nanjing)	NA	ALL	Hospital	152 cases (107 males) including 63 SC and 89 AC	152 controls without lung disease matched for gender, age (±5)	82/152	79/152	85/152	58/152		
39*	Yang XHR 2004 [92]	Heilongjiang (Shenyang)	1985.9–1987.9	ALL	Population	200 cases	144 healthy controls, matched by age	108/186	75/139				
40*	Moira CY 2004 [93]	Hong Kong	1999.7–2001.6	ALL	Population	229 cases (127 AC and 38 SC)	197 healthy controls, significantly younger	130/229	117/197	143/229	102/197		
41*	Lan Q 2004 [94]	Yunnan (Xuanwei)	1995.3–1996	NA	Population	122 cases	122 controls matched by age, gender and smoking status	82/122	60/122	73/122	64/122		
42	Gu YF 2004 [95]	Beijing	NA	ALL	Hospital and Population	180 cases (124 males) including 52 SA, 66 AC, 29 SCLC, 11 mixed style and 22 others	224 controls (117 controls with lung benign disease and 107 healthy controls), equally comparable in gender, age, ethnicity	101/180	102/224				
43	Dong CT 2004 [96]	Sichuan	2001.1–2001.11	ALL	Hospital	82 cases	91 respiratory system disease controls without tumor, comparable in age, gender and ethnicity	48/82	36/91				
44	Luo CL 2004 [97]	Guangzhou	NA	ALL	Population	63 cases (49 males) including 24 SC, 28 AC, 7 SCLC and 4 others	47 healthy controls, comparable in age, gender and ethnicity	45/63	24/47				
45	Cao YF 2004 [98]	Hunan	NA	ALL	Population	104 cases	205 controls, comparable in age, gender	65/104	95/205	69/104	87/205	43/104	46/205
46	Chen SD 2004 [99]	Guangdong	2000–2001	NA	Hospital	91 cases	91 controls, comparable in age and gender	56/91	51/91				
47	Huang XH 2004 [100]	Guangdong (Guangzhou)	2000.10–2002.1	ALL	Hospital and Population	91 cases including 54 SC, 31 AC and 6 SCLC	138 control (91 hospital patients and 47 healthy controls), matched by age, gender, and residence	56/91	73/138				
48	Ye WY 2004 [101]	Guangdong (Guangzhou)	2000.10–2002.1	ALL	Hospital	58 cases (age in 35–85 years, 38 males and 20 females)	62 controls without respiratory disease and tumor (age in 35–85 years, 42 males),comparable in gender and age	35/58	29/62				
49*	Wang JW 2003 [102]	Beijing	1998–2000	ALL	Population	112AC cases	119 healthy controls matched for age and gender	Δ69/112	Δ60/119	53/112	54/119	36/112	29/119
50*	Wang JW 2003 [103]	Beijing/Tianjin	1998–2000	ALL	Population	164 AC cases (112 in Beijing, 52 in Tianjin)	181 cancer-free controls matched for gender and age	97/164	90/181				
51	Chen LJ 2003 [104]	Anhui (Wuhu)	NA	ALL	Population	38 cases	99 healthy controls, comparable in age and gender	24/38	57/99				
52	Li WY 2003 [105]	Beijing	NA	ALL	Hospital	217 cases	200 non-cancer controls, comparable in age, gender and township of residence	127/217	95/200				
53*	Lu WF 2002 [106]	Beijing and surrounding regions	1997.1–2000.12	ALL	Population	314 cases (177 SC and 137 AC)	320 normal controls, matched for age, gender and smoking status	158/314	155/314				
54a	Qiao GB 2002 [107]	Guangzhou	1997.1–1999.12	ALL	Hospital	213 cases (106 SC, 62 AC, 45 others)	64 with lung benign disease	130/213	31/64				
54b	Qiao GB 2002 [107]	Guangzhou	1997.1–1999.12	ALL	Population	213 cases (106 SC, 62 AC, 45 others)	135 healthy cases	130/213	64/135				
55	Zhang LZ 2002 [108]	Jiangsu (Xuzhou)	1999.3–2000.10	ALL	Hospital	65 cases (age 59.4±8.4 years, 56 males) including 34 SC, 25 AC, 2 SCLC and 4 others	60 controls (age 55.6±7.5 years, 54 males)	41/65	27/60				
56	Shi Y 2002 [109]	Hubei	NA	ALL	Hospital	120 cases	120 noncancer controls, comparable in age and gender in Han population	74/120	53/120				
57	Zhang JK 2002 [110]	Guangdong (Guangzhou)	1999.1–2000.5	ALL	Population	42 females cases	55 healthy females match by age in Han population	Δ28/42	Δ30/55	Δ19/42	Δ21/55	12/42	10/55
58	Zhang JK 2002 [111]	Guangdong (Guangzhou)	1999.1–2000.5	ALL	Population	161 cases	165 healthy controls, comparable in age and gender	94/161	92/165	74/161	72/165		
59	Xin Y 2002 [112]	Yunnan	NA	NA	Population	56 cases	99 healthy controls	43/56	65/99				
60*	Cheng YW 2001 [113]	Taiwan	NA	NA	Hospital	62 nonsmoking cases	20 noncancer controls with lung disease and comparable in age and gender	25/62	10/20				
61*	Chen SQ 2001[114]	Jiangsu	NA	ALL	Population	106 cases	106 healthy controls matched for gender and age	56/106	39/106				
62*	Stephanie J London 2000 [115]	Shanghai	1986.1–1997.3	PARTIAL	Population	234 cases	714 controls matched for age and residential township	122/232	427/710	134/232	426/710	85/232	275/710
63*	Cheng YW 2000 [116]	Taiwan	NA	NA	Hospital	73 cases	33 noncancer controls with lung cancer and comparable in age, gender and smoking status	34/73	17/33				
64	Lan Q 1999 [117]	Yunnan (Xuanwei)	1994.7–1995.11	PARTIAL	Population	86 cases	86 controls equally comparable in age and gender	56/86	38/86	52/86	52/86		
65a*	Gao Y 1999 [118]	Guangdong (Guangzhou)	1996.11–1997.3	ALL	Population	59 cases (26 AC, 23 SC and 10 mixed style)	73 healthy controls in Han population matched by age and gender	34/59	36/73				
65b*	Gao Y 1999 [118]	Guangdong (Guangzhou)	1996.11–1997.3	ALL	Hospital	59 cases (26 AC, 23 SC and 10 mixed style)	59 free-cancer controls without hereditary disease matched by age and gender	34/59	29/59				
66	Chen SQ 1999 [119]	Jiangsu	NA	NA	Population	68 cases	105 healthy controls	39/68	42/105				
67	Gao JR1998 [120]	Guangdong	1995.11–1996.4	ALL	Population	46 cases	70 controls equally comparable in age, gender, ethnicity and residential township	27/46	25/70				
68a	Qu YH 1998[121]	Shanghai	NA	NA	Population	100 female cases (age 60.18±12.18 years)	95 healthy controls (age 60.48±12.29 years)	56/100	49/94				
68b	Qu YH 1998 [121]	Heilongjiang (Haerbin)	NA	NA	Population	82 female cases (age 47.99±12.17)	85 healthy controls (age 47.36±11.17 years)	46/82	45/85				
69*	Sun GF 1997 [122]	Liaoning	1992.1–1994.12	ALL	Population	207 cases including 86 SC, 68 AC and 53 SCLC	364 controls	147/207	186/364				
70a*	Ge H 1996 [123]	Hong Kong	1989–1994	ALL	Population	98 NSCLC cases (61 males), including 66AC, 26 SCC, 6 others)	25 healthy controls	59/89	16/25				
70b*	Ge H 1996 [123]	Hong Kong	1989–1994	ALL	Hospital	89 NSCLC cases	28 bronchiectasis patients	59/89	19/28				
71	Sun GF 1995 [124]	NA	NA	ALL	Population	175 cases	104 healthy controls	125/175	54/104				

Pathologic diagnosis^¶^: ALL means that all lung cancer cases were confirmed by pathologic diagnosis; PARTIAL means that partial cases were confirmed by pathologic diagnosis; NA means that relative data were not available in original studies.

SC: Squamous Carcinoma; AC: Adenocarcinoma; SCLC: Small Cell Lung Carcinoma; NSCLC: Non-small-cell Lung Carcinoma. *: Articles published in English.

^△^ : These data were omitted because of a larger sample from the same studied population by the same research group.

a/b: A study with two distinct controls encompassed population-based and hospital-based could been analyzed, respectively.

**Table 2:** The contextual details of subgroup analysis included in this meta-analysis.

No.	Study	Material used for detecting GSTs genotype	Combined evaluation of other genes	Gene	*CYP1A1* (Msp1) HWE	Null *GSTs* genotype (%)	Non-smokerФ		smoker		Study type	Quality score^ζ^
							Case	Control	Case	Control		
	2012											
1	Liu DZ et al[54]	WBC	NA	*GSTM1*	NA	29.7	42/105	52/175	103/255	55/185	EG	8
2	Wang N et al[55]	WBC	*CYP1A1,mEH, XRCC1*	*GSTM1/GSTT1*	YES	44.1/39.1	NA	NA	NA	NA	EG	8
3	Li WY et al[56]	WBC	*CYP1A1,CYP2E1, CYP2D6*	*GSTM1*	YES	47.0	55/96	70/135	72/121	23/63	EG	8
4	Chen CM et al[57]	WBC	*CYP1A1*	*GSTM1*	YES*	55.3	34/54	47/76	89/146	63/113	EG	7
5	Yao ZG et al[58]	WBC	*NA*	*GSTM1*	NA	45.3	29/45	38/78	67/105	30/72	EG	8
6	Liu JN et al[59]	WBC	*NA*	*GSTT1*	NA	41.5	26/51	38/85	31/49	18/50	EG	6
7	Han RL et al[60]	WBC	*NA*	*GSTM1*	NA	41.6	26/45	54/115	60/83	35/99	EG	5
	2011											
8	Jin YT et al [61]	WBC	*CYP1A1*	*GSTM1*	NO/YES*	37.7	OR 95% CI = 0.76(0.18–3.17)		OR 95% CI = 2.11(0.66–6.88)		EG	6
9	Ai C et al[62]	WBC	*NA*	*GSTM1*	NA	46.0	NA	NA	NA	NA	EG	8
10	Zhang JQ et al[63]	WBC	*NA*	*GSTM1*	NA	44.0	13/22	9/24	21/28	13/26	EG	7
11	Du GB et al[64]	WBC	*NA*	*GSTM1*	NA	56.8	32/49	46/82	41/76	23/43	EG	6
12	Li Y et al[65]	cases: BALF cells, controls: WBC	*CYP1A1*	*GSTM1*	YES/YES*	44.2	20/27	28/64	43/76	33/74	EG	7
13	Bai TY et al[66]	NA	*NA*	*GSTT1*	NA	44.4	24/63	20/71	32/76	30/40	NA	4
	2010											
14	Jin YT et al[67]	WBC	*CYP1A1*	*GSTM1*	NA	52.7	25/37	28/63	70/113	51/87	EG	7
15	Zheng DJ et al[68]	WBC	*NA*	*GSTM1*	NA	57.0	NA	NA	NA	NA	EG	8
16	Zhu XX et al[69]	WBC	*CYP1A1*	*GSTM1*	YES/YES*	45.0	NA	NA	NA	NA	EG	8
17	Fan J et al[70]	WBC	*NA*	*GSTM1*	NA	55.0	23/32	22/40	17/26	11/20	EG	7
				*GSTT1*	NA	48.3	20/32	21/41	18/26	8/19	EG	
	2009											
18	Chang FH et al[71]	WBC	*CYP1A1*	*GSTM1*	NA	47.9	60/97	101/145	92/166	25/118	EG	7
	2008											
19	Chen H et al[72]	WBC	*CYP1A1*	*GSTM1*	NO	54.2	26/39	126/246	73/119	120/208	EG	8
20	Liu Q et al[73]	WBC	*CYP1A1*	*GSTM1*	NO	45.6	NA	NA	NA	NA	EG	8
21Ψ	Qi XS et al[74]	WBC	*NA*	*GSTT1*	NA	37.5	0/5	4/13	17/47	23/59	EG	7
				*GSTM1*	NA	56.9	NA	NA	NA	NA		
22	Xia Y et al[75]	WBC	*CYP1A1*	*GSTM1*	YES	37.5	NA	NA	NA	NA	EG	6
	2007											
23	Gu YF et al[76]	WBC	*CYP1A1,2D6,2E1*	*GSTM1*	NA	47.5	NA	NA	NA	NA	EG	7
24‡	Wang YS et al[77]	WBC/Adjacent normal tissue	*NA*	*GSTM1*	NA	53.2	OR 95% CI = 1.07(0.19–5.96)	OR = 1	OR 95% CI = 1.57(0.48–5.27)	OR 95% CI = 1.29(0.37–4.68)	EG	7
25	Lei FM et al[78]	WBC	*NA*	*GSTM1*	NA	55.3	NA	NA	NA	NA	EG	8
	2006											
26	Chang FH et al[79]	WBC	*CYP1A1*	*GSTM1*	NA	47.9	44/62	62/96	62/101	16/67	EG	6
27	Chen HC et al[80]	WBC	*NAT2,GSTP1*	*GSTM1*	NA	45.2	NA	NA	NA	NA	EG	7
				*GSTT1*	NA	43.1	NA	NA	NA	NA	EG	
28	Li Y et al[81]	case: BALF cells control: WBC	*CYP1A1*	*GSTM1*	YES/YES*	44.1	19/26	28/63	41/72	32/73	EG	8
29	Yao W et al[82]	case: lung cancer tissue/control: WBC	*NA*	*GSTM1*	NA	42.1	NA	NA	NA	NA	NEG	NA
				*GSTT1*	NA	50.5	NA	NA	NA	NA		NA
30	Qian BY et al[83]	NA	*CYP1A1*	*GSTM1*	YES	49.1	15/23	22/46	54/85	31/62	NEG	NA
31	Wang QM et al[84]	WBC	*CYP2C9*	*GSTM1*	NA	45.2	10/19	7/19	30/37	12/23	EG	4
32	He DX et al[85]	WBC	*NA*	*GSTT1*	NA	63.0	NA	NA	NA	NA	EG	5
	2005											
33	Chan EC et al[86]	case: uninvolved lung tissue/control: WBC	*GSTP1, MPO etc.*	*GSTM1*	NA	56.2	NA	NA	NA	NA	EG	5
34	Yuan TZ et al[87]	WBC	*NA*	*GSTT1*	NA	38.2	12/52	39/100	70/98	19/52	EG	7
35	Li DR et al[88]	WBC	*CYP2E1*	*GSTM1*	NA	40.9	22/36	17/50	35/63	10/16	EG	5
36	Ye WY et al[89]	WBC	*NA*	*GSTM1*	NA	53.2	NA	NA	NA	NA	EG	6
37	Chou YC et al[90]	WBC	*NA*	*GSTM1*	NA	65.0	NA	NA	NA	NA	EG	8
	2004											
38	Liang GY et al[91]	WBC	*CYP1A1, 2E1, GSTP1 etc.*	*GSTM1/GSTT1*	YES	52.0/38.2	NA	NA	NA	NA	EG	6
39	Yang XHR et al[92]	WBC	*CYP1A1*	*GSTM1*	NA	54.0	OR 95% CI = 1.05(0.56–2.00)		OR 95% CI = 1.61(0.80–3.25)		EG	7
40	Moira CY et al[93]	WBC	*GSTP1*	*GSTM1*	NA	59.4	NA		NA		EG	6
				*GSTT1*	NA	51.8	OR ^a^ 95% CI = 2.18(1.21–3.94)		NA			
41	Lan Q et al[94]	buccal cells	*p53*	*GSTM1/GSTT1*	NA	49.2/52.5	NA	NA	NA	NA	NEG	NA
42	Gu YF et al[95]	WBC	*CYP1A1, 2D6, 2E1*	*GSTM1*	NA	45.5	OR 95% CI = 2.01(0.53,8.22)		OR 95% CI = 5.50(1.43,22.89)^I^		EG	5
43	Dong CT et al[96]	WBC	*CYP1A1*	*GSTM1*	NA	39.6	NA	NA	NA	NA	EG	7
44	Luo CL et al[97]	WBC	*p53*	*GSTM1*	NA	51.1	NA	NA	NA	NA	EG	6
45	Cao YF et al[98]	WBC	*NA*	*GSTM1/GSTT1*	NA	46.3/42.4	NA	NA	NA	NA	EG	7
46	Chen SD et al[99]	WBC	*CYP2E1*	*GSTM1*	NA	56.0	25/36	31/59	31/55	18/32	EG	7
47	Huang XH et al[100]	WBC	*NA*	*GSTM1*	NA	52.9	25/36	39/76	31/55	34/62	EG	7
48	Ye WY et al[101]	WBC	*NA*	*GSTM1*	NA	46.8	NA	NA	NA	NA	EG	7
	2003											
49Ψ	Wang JW et al[102]	WBC	*GSTP1*	*GSTM1*	NA	50.4	40/64	36/71	29/48	24/48	EG	6
				*GSTT1*	NA	49.7	30/64	27/71	23/48	27/48		
50	Wang JW et al[103]	WBC	*CYP2E1, 1A1*	*GSTM1*	YES	57.6	53/94	52/105	44/70	38/76	EG	8
51	Chen LJ et al[104]	WBC	*NA*	*GSTM1*	NA	47.5	8/13	36/63	16/25	21/36	EG	7
52	Li WY et al[105]	WBC	*CYP1A1,2E1, 2D6*	*GSTM1*	YES	50.4	55/96	70/135	72/121	25/65	EG	6
	2002											
53	Lu WF et al[106]	case: “normal” tissue adjacent to tumor/control: WBC	*MPO*	*GSTM1*	NA	49.4	54/111	154/298	104/203	156/330	EG	8
54a	Qiao GB et al[107]	case: tumor tissue/control: benign lung tissue	*NA*	*GSTM1*	NA	48.4	NA	NA	NA	NA	EG	7
54b	Qiao GB et al[107]	case: tumor tissue/control: WBC	*NA*	*GSTM1*	NA	47.4	NA	NA	NA	NA	EG	6
55	Zhang LZ et al[108]	case: lung cancer tissue/control: WBC	*CYP1A1*	*GSTM1*	NA	45.0	8/14	14/28	33/51	13/32	NEG	NA
56	Shi Y et al[109]	WBC	*CYP2E1*	*GSTM1*	NA	44.2	NA	NA	NA	NA	EG	6
57Ψ	Zhang JK et al[110]	WBC	*NA*	*GSTM1*	NA	54.5	28/38	23/44	NA	NA	Female/EG	7
				*GSTT1*	NA	38.2	18/38	18/44	NA	NA		
58	Zhang JK et al[111]	WBC	*NA*	*GSTM1*	NA	55.8	39/57	52/100	NA	NA	EG	7
				*GSTT1*	NA	43.6	27/57	44/100	NA	NA		
59	Xin Y et al[112]	WBC	*NA*	*GSTM1*	NA	65.7	NA	NA	NA	NA	EG	4
	2001											
60	Cheng YW et al[113]	case: normal tissue surrounding lung tumor/control: NA	*NA*	*GSTM1*	NA	50.0	NA	NA	NA	NA	NEG	NA
61	Chen SQ et al[114]	WBC	*CYP1A1*	*GSTM1*	NA	36.8	NA	NA	42/80	29/80	EG	7
	2000											
62	Stephanie J London et al[115]	WBC	*NA*	*GSTM1/GSTT1*	NA	60.1/60.0	NA	NA	NA	NA	EG	7
63	Cheng YW et a[116]l	non-tumorous area cell	*CYP1A1*	*GSTM1*	YES	51.5	NA	NA	NA	NA	NEG	NA
	1999											
64	Lan Q et al[117]	buccal cells	*NA*	GSTM1/GSTT1	NA	44.2/60.5	NA	NA	NA	NA	NEG	NA
65a	Gao Y et al[118]	NA	*NA*	*GSTM1*	NA	49.3	14/21	26/51	20/38	10/22	EG	8
65b	Gao Y et al[118]	NA	*NA*	*GSTM1*	NA	49.2	14/21	20/34	20/38	9/25	EG	7
66	Chen SQ et al[119]	WBC	*CYP1A1*	*GSTM1*	NA	40.0	NA	NA	NA	NA	EG	5
	1998											
67	Gao JR et al[120]	WBC	*CYP2D6*	*GSTM1*	NA	35.7	NA	NA	NA	NA	EG	8
68a	Qu YH et al[121]	WBC:	*CYP1A1*	*GSTM1*	YES	52.1	56/100	49/94	NA	NA	Female/EG	5
68b	Qu YH et al[121]	WBC	*CYP1A1*	*GSTM1*	YES	52.9	46/82	45/85	NA	NA	Female/EG	4
	1997											
69	Sun GF et al[122]	WBC	*NA*	*GSTM1*	NA	51.1	49/67	97/191	98/140	89/173	EG	6
	1996											
70a	Ge H et al[123]	case: normal lung tissue, WBC/control: WBC	*L-myc*	*GSTM1*	NA	64.0	NA	NA	NA	NA	EG	6
70b	Ge H et al[123]	case: normal lung tissue, WBC/control: WBC	*L-myc*	*GSTM1*	NA	67.9	NA	NA	NA	NA	EG	5
	1995											
71	Sun GF et al[124]	WBC	*NA*	*GSTM1*	NA	51.9	36/52	38/74	89/123	16/30	EG	5

HWE: Hardy-Weinberg Equilibrium; WBC: White blood cells; BALF: bronchoalveolar lavage fluid; NA: not available.

*: The HWE test results of *CYP1A1* Msp1 that could be calculated were shown in the table, and the items with * meant the result that had been reported in the articles.

^Φ^ : Due to different setting of smoking status in papers, people who had smoked were calculated as smokers.

OR^a^: Adjusted OR. ED: Epidemiological Design; NED: Non-epidemiology Design; WBC: blood, White blood cell lymphocytes, and serum. ^ζ^: Newcastle-Ottawa Scale (NOS).

^‡^ : The OR 95% CI was captured from logistic analysis; I: Heavy-smoker; a: healthy control; b: hospital control.

^Ψ^ : The *GSTM1* data of this study was omitted because of a bigger sample in the other study published in the same year.

### 4. Statistical analysis

(1) The pooled ORs and 95% CIs were determined by the Z test, *P*≤0.05 was considered statistically significant. (2) Statistical heterogeneity among studies was assessed by *Q* and *I^2^* statistics [50]. In heterogeneity tests, when *P*≤0.1, a random-effects model was used; when *P*>0.1, a fixed-effects model was performed [51]. Meanwhile, if *I^2^*≥50%, 50%>*I^2^*≥25% or *I^2^*<25%, we identified the studies as high, middle or low heterogeneity, respectively. (3) Sensitivity analysis was performed by removing one study at a time to calculate the overall homogeneity and effect size; the Galbraith plot was also performed to examine the possible distinct articles. (4) The possible reasons for heterogeneity between studies were investigated by subgroup analyses. Nine subgroups were analyzed as follows: histopathological classification (SC, AC or SCLC), geographical location (North, Northeast, Northwest, East, Central, South, or Southwest of China) (See Figure S1), smoking status (smoker *vs*. non-smoker), *CYP1A1*(Msp1) polymorphisms, case number (<100 *vs*. ≥100), source of controls (population-based *vs*. hospital-based), research design (epidemiological design *vs*. non-epidemiological design), test material (white blood cells, involved tissues or other cells, or not available) and quality score (4–5, 6, 7–8). The last five items listed above were used to assess the study quality. (5) Cumulative meta-analysis was used to explore any significant changes in the variation of sample size or publication year. (6) Publication bias was investigated by the Begg's test [52], Egger's linear regression test and Trim and Fill test [53]. (7) All analyses were performed with the software Stata version 12.0 (StataCorp LP, College Station, Texas, USA), and all *P* values were two sided.

## Results

### 1. Study selection and study characteristics

**Figure 1:**
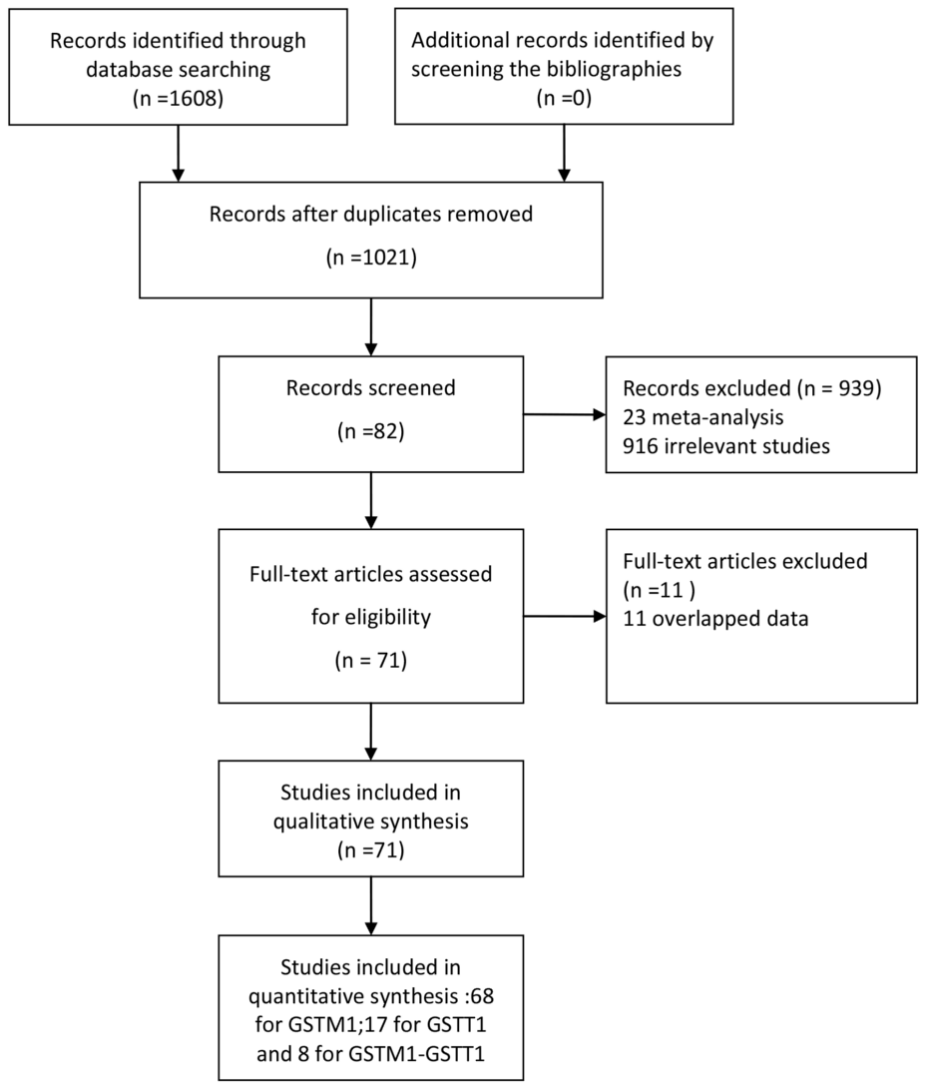
Study flow chart.

We ultimately identified a total of 71 articles [54]–[124] reporting the relationship between *GSTM1* and/or *GSTT1* genetic polymorphisms and lung cancer risk from both Chinese and English databases (Figure 1). There were 68 studies about *GSTM1* (8649 cases and 10380 controls) [54]–[58], [60]–[65], [67]–[73], [75]–[84], [86], [88]–[101], [103]–[109], [111]–[124] published between 1995 and 2012, 17 studies about *GSTT1* (2109 cases and 3031 controls) [55], [59], [66], [70], [74], [80], [82], [85], [87], [91], [93], [94], [98], [103], [111], [115], [117] between 1999 and 2012 and 8 studies about both *GSTM1* and *GSTT1* (775 cases and 1495 controls) [70], [74], [80], [82], [98], [102], [110], [115] between 2000 and 2010.

**Figure 2:**
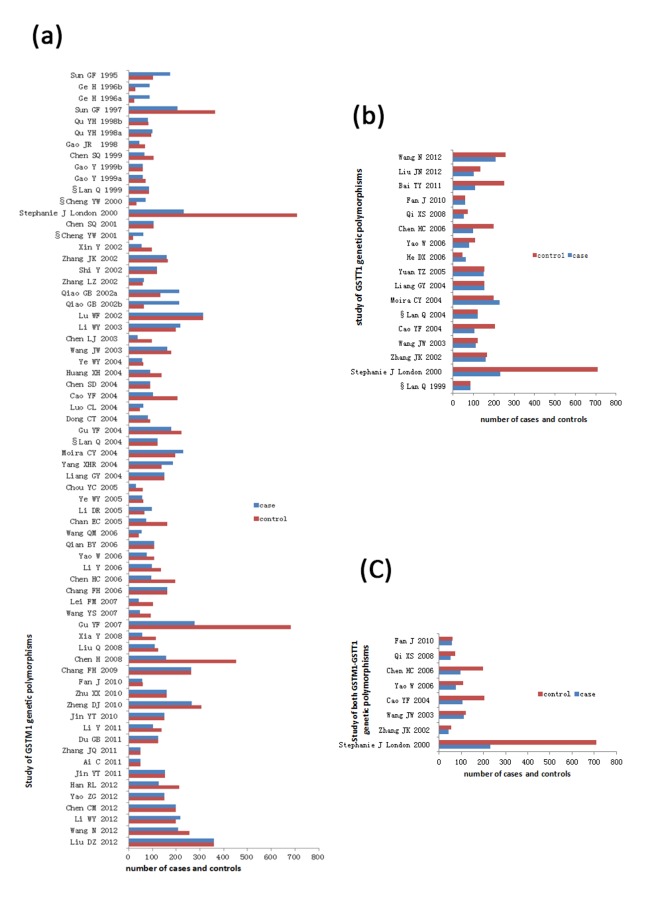
Cases and controls of 71 published studies included in this meta-analysis. (a) 68 literatures about *GSTM1* genetic variants and lung cancer risk; (b) 17 literatures about *GSTT1* genetic variants and lung cancer risk; (c) 8 literatures about *GSTM1*-*GSTT1* genetic variants dual null genotype and lung cancer risk.

Most studies were published in Chinese (49/68 of *GSTM1* studies, 13/17 of *GSTT1*, and 5/8 of both *GSTM1* and *GSTT1*). According to our criterion, 61 (89.7%) studies of *GSTM1*, 13 (76.5%) of *GSTT1*, and 7 (87.5%) of both *GSTM1* and *GSTT1* were evaluated as epidemiological designs. In both control and case groups, 50 (73.5%) studies of *GSTM1*, 13 (76.5%) of *GSTT1* and 7 (87.5%) of both *GSTM1* and *GSTT1* used white blood cells for GSTs genotype detection. The rest of the studies used adjacent lung tissue, tumor tissue, BALF cells or buccal cells, etc., for GSTs genotype detection in cases or controls. Only two studies reported the HWE test results for the *GSTM1* or *GSTT1* and satisfied HWE [57], [81]. In the eligible studies, the null genotype frequency of *GSTM1* and *GSTT1* ranged from 29.7% to 67.9% (Mean = 49.5%) and 37.5% to 63.0% (Median = 44.4%), respectively. The *CYP1A1* (Msp1) polymorphisms satisfied the HWE in the controls of 15 (68%) studies about *GSTM1* and *CYP1A1* (Msp1). More details are shown in Table 1, Table 2 and Figure 2.

### 2. Synthesis results of all studies

**Figure 3:**
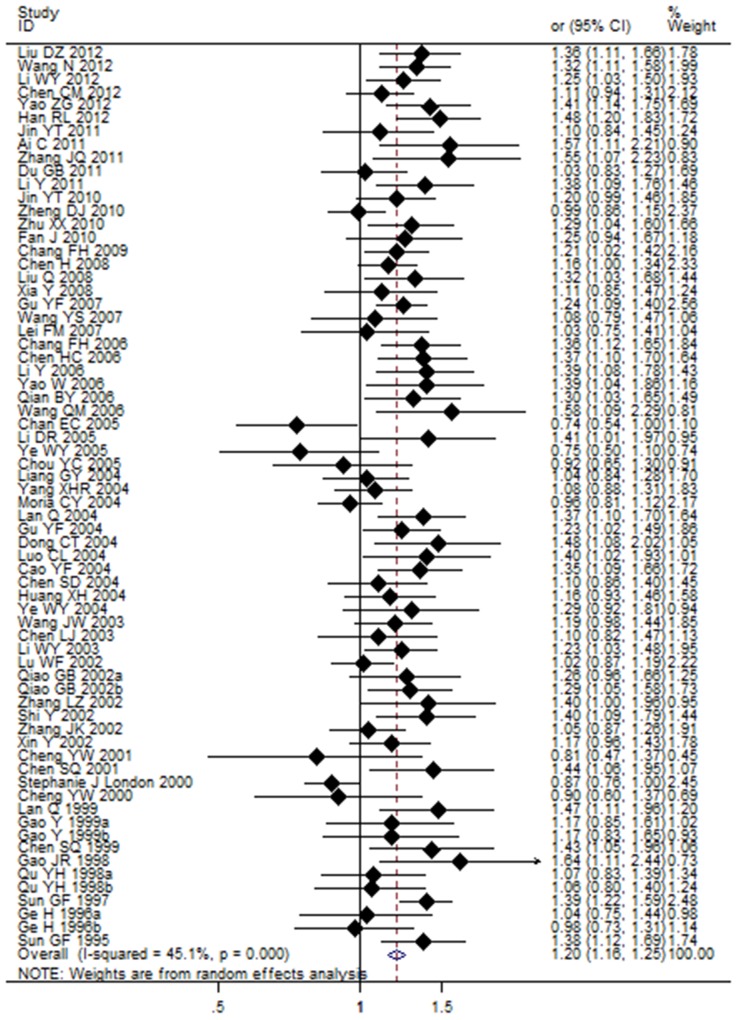
Association between *GSTM1* null genotype and lung cancer susceptibility analyzed by the Forest plot. The Forest plots of pooled OR with 95% CI (Null genotype *vs*. Present genotype; OR = 1.20, 95% CI: 1.16 to 1.25; Random-effects model, *P*<0.001).

**Figure 4:**
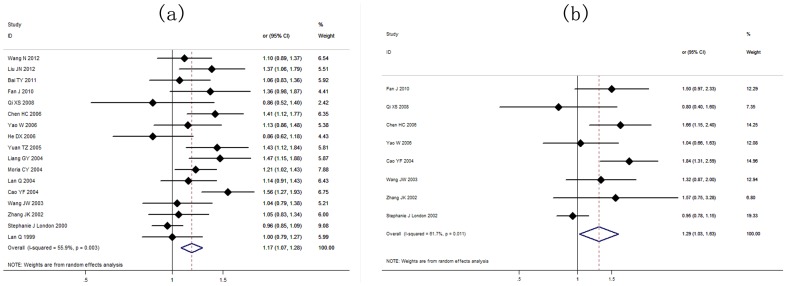
(a) Association between *GSTT1* null genotype and lung cancer susceptibility analyzed by the Forest plot. The Forest plots of pooled OR with 95% CI (Null genotype *vs*. Present genotype; OR = 1.17, 95% CI: 1.07 to 1.28; Random-effects model, *P*<0.001). (b) Association between *GSTM1-GSTT1* dual-null genotype and lung cancer susceptibility analyzed by the Forest plot The Forest plots of pooled OR with 95% CI (Dual-null genotype *vs*. Present genotype; OR = 1.29, 95% CI: 1.03 to 1.63; Random-effects model, *P*<0.001).

The results showed a significant association between the *GSTM1* null genotype and lung cancer risk in the Chinese population under the random-effects model (OR = 1.20, 95% CI: 1.16 to 1.25, *I^2^* = 45.1%, *P*<0.001) (Table 3). The random-effects model showed that the *GSTT1* null genotype was significantly correlated with lung cancer risk in the Chinese population (OR = 1.17, 95% CI: 1.07 to 1.28, *I^2^* = 55.9%, *P*<0.001) (Table 4). Further analyses showed that dual-null genotype of *GSTM1-GSTT1* had a significant higher association with lung cancer risk (OR = 1.29, 95% CI: 1.03 to 1.63, *I^2^* = 61.7%, *P* = 0.011) (Table 5). Risk estimation for each study is shown in the Forest plots in Figure 3, Figure 4a and Figure 4b.

**Table 3:** Subgroup analysis of the association between *GSTM1* null genotype and lung cancer risk.

Polymorphism	Null *vs*. Present	No. of studies (cases/controls)	Odds ratio		M	Heterogeneity		*P_E_*
			OR[95%CI]	*POR*		*I^2^* (%)	*PH*	
*GSTM1*	All studies	68(8649/10380)	1.20[1.16,1.25]	<0.001	R	45.1	<0.001	0.245
	subgroup analyses by histopathology classification.							
	Squamous Carcinoma	14(1088/3218)	1.20[1.12,1.27]	<0.001	F	19.5	0.241	0.790
	Adenocarcinoma	13(1060/3093)	1.14[1.03, 1.26]	0.008	R	50.3	0.020	0.491
	Small Cell Lung Carcinoma	5(179/1853)	1.29[1.13,1.47]	<0.001	F	38.7	0.163	0.313
	subgroup analyses by geographical location^¤^							
	North China	11(2320/2792)	1.19[1.13,1.25]	<0.001	F	35.6	0.114	0.099
	Northeast of China	4(835/948)	1.24[1.07,1.43]	0.004	R	54.1	0.088	0.252
	Northwest of China	1(58/116)	1.11[0.85,1.47]	0.442	R	@	@	@
	East China	16(1745/2615)	1.11[1.02,1.20]	0.011	R	40.8	0.045	0.387
	Central China	8(968/1319)	1.35[1.25,1.47]	<0.001	F	0	1.000	0.050
	South China	15(1577/1276)	1.13[1.05,1.21]	<0.001	F	25.5	0.174	0.221
	Southwest of China	9(737/904)	1.21[1.04,1.40]	0.011	R	61.6	0.008	0.646
	subgroup analyses by smoking status							
	smoker•	32(NA/NA)	1.34[1.23,1.47]	<0.001	R	53.8	<0.001	0.008‡
	non-smoker	35(NA/NA)	1.20[1.13,1.26]	<0.001	F	14.6	0.226	0.052
	subgroup analyses by *CYP1A1*(Msp1)							
	wt/wt	11(578/961)	1.17[1.06,1.30]	0.002	F	0	0.891	0.678
	wt/mt	10(732/926)	1.23[1.12,1.35]	<0.001	F	12.7	0.326	0.631
	mt/mt	6(203/167)	1.34[1.13,1.59]	0.001	F	0	0.979	0.010‡
	subgroup analyses by number of case							
	<100	32(2152/2576)	1.20[1.12,1.28]	<0.001	R	35.5	0.026	0.582
	≥100	36(6497/7804)	1.20 [1.15,1.26]	<0.001	R	52.6	<0.001	0.024‡
	subgroup analyses by source of control							
	Population-based	45(5883/7304)	1.21[1.15,1.27]	<0.001	R	53.3	<0.001	0.026
	Hospital-based	20(2216/2030)	1.20[1.13,1.27]	<0.001	F	30.1	0.101	0.150
	Mixed-based	3(550/1046)	1.22[1.11,1.35]	<0.001	F	0	0.893	0.603
	subgroup analyses by research design							
	Epidemiological study	61(8056/9844)	1.20[1.15,1.24]	<0.001	R	46.4	<0.001	0.175
	Non-epidemiological study	7(593/536)	1.30[1.16,1.45]	<0.001	F	19.1	0.284	0.046‡
	subgroup analyses by test material							
	White blood cells	50(6697/8616)	1.21[1.16,1.26]	<0.001	R	46.7	<0.001	0.069
	Involved tissue or cell†	15(1726/1524)	1.17[1.06,1.30]	0.003	R	52.2	0.009	0.554
	Not available	3(226/240)	1.23[1.04,1.45]	0.014	F	0	0.822	0.115
	subgroup analyses by quality score^¶^ (Epidemiological study)							
	4–5	11(1108/1223)	1.20[1.07,1.36]	0.002	R	57	0.010	0.606
	6	13(1948/1960)	1.15[1.06,1.26]	0.002	R	52.8	0.013	0.240
	7–8	44(5593/7197)	1.21[1.16,1.27]	<0.001	R	40.9	0.003	0.023‡

¤: Geographical locations of China were divided into 7 parts: Northeast of China (Jilin province, Liaoning province, Heilongjiang province), North China (Beijing city, Tianjin city, Heber province, Shanxi Province (Taiyuan), Inner Mongolia), East China (Shanghai city, Anhui province, Jiangxi province, Jiangsu province, Zhejiang province, Fujian province, Shandong province, Taiwan), Central China (Henan province, Hubei province, Hunan province), South China (Guangdong province, Hainan province, Guangxi Zhuang Autonomous Region, Hongkong), Southwest of China (Chongqing City, Guizhou province, Sichuan Province, Yunnan Province, Tibet), Northwest of China (Shanxi province (xi'an), Gansu province, Ningxia Hui Autonomous Region, Xinjiang Uyghur autonomous region).

M: model of meta-analysis; R: random-effects model; F: fixed-effects model.*P_H_*: *p* value of heterogeneity test. *P_E_*:*p* value of Egger's test.*P_OR_*: *P*<0.001 replace *P* = 0.000 and *P* less than 0.001. @: *p* values could not be calculated.

^‡^ : the publication bias was detected in this group. ^¶^: Newcastle-Ottawa Scale (NOS).

^†^ : test materials of case or control was from the normal lung tissues, BALF cells, buccal cells or lung cancer tissue.

^•^ :the study of Wang YS et al was not included because of the unavailable data.

**Table 4:** Subgroup analysis of the association between *GSTT1* null genotype and lung cancer risk.

Polymorphism	Null *vs*. Present	No.ofstudies (cases/controls)	Odds ratio		M	Heterogeneity		*P_E_*
			OR[95%CI]	*POR*		*I^2^*(%)	*PH*	
*GSTT1*	All studies	17(2109/3031)	1.17[1,07,1.28]	<0.001	R	55.9	0.003	0.510
	subgroup analyses by histopathology classification							
	Squamous Carcinoma	5(240/680)	1.38[1.20,1.59]	<0.001	F	38.9	0.162	0.222
	Adenocarcinoma	4(389/620)	1.23[1.08,1.40]	0.001	F	0	0.546	0.993
	Small Cell Lung Carcinoma	NA	NA	NA	NA	NA	NA	NA
	subgroup analyses by geographical location^¤^							
	North China	2(218/369)	1.05[0.88,1.27]	0.576	F	0	0.922	@
	Northeast of China	NA	NA	NA	NA	NA	NA	NA
	Northwest of China	1(53/72)	0.86[0.52,1.40]	0.534	@	@	@	@
	East China	2(384/862)	1.17[0.77,1.77]]	0.454	R	88.9	0.003	@
	Central China	4(487/765)	1.30[1.09,1.54]	0.003	R	55.4	0.081	0.485
	South China	3(448/422)	1.17[1.03,1.33]	0.013	F	0	0.440	0.876
	Southwest of China	4(419/406)	1.10[0.90,1.35]	0.341	R	59.4	0.060	0.487
	subgroup analyses by smoking status							
	smoker	6(344/268)	1.15[0.73,1.81]	0.541	R	85.8	<0.001	0.301
	non-smoker	8(NA/NA)	1.16[0.93,1.45]	0.187	R	41.7	0.100	0.596
	subgroup analyses by number of case							
	<100	6(432/568)	1.11[0.94,1.32]	0.221	R	49.8	0.077	0.327
	≥100	11(1677/2463)	1.19[1.08,1.33]	0.001	R	61.8	0.004	0.094
	subgroup analyses by source of control							
	Population-based	14(1798/2557)	1.17[1.07,1.29]	0.001	R	57.7	0.004	0.284
	Hospital-based	3(311/474)	1.15[0.86,1.54]	0.335	R	62.2	0.071	0.587
	subgroup analyses by research design							
	Epidemiological study	13(1718/2466)	1.20[1.07,1.34]	0.001	R	64.9	0.001	0.464
	Non-epidemiological study	3(285/315)	1.09[0.95,1.26]	0.214	F	0	0.695	0.971
	subgroup analyses by test material							
	White blood cells	13(1718/2466)	1.20[1.07,1.34]	0.001	R	64.9	0.001	0.464
	Involved tissue or cell†	3(285/315)	1.09[0.95,1.26]	0.214	F	0	0.695	0.971
	Not available	1(106/250)	1.06[0.83,1.36]	0.628	@	@	@	@
	subgroup analyses by quality score^¶^ (Epidemiological study)							
	4–5	4(366/525)	1.07[0.94,1.22]	0.310	F	0	0.510	0.158
	6	4(593/603)	1.26[1.13,1.41]	<0.001	F	23.1	0.272	0.860
	7–8	9(1150/1903)	1.18[1.03,1.36]	0.020	R	69.6	0.001	0.380

¤: geographical locations of China were divided into 7 parts: Northeast of China (Jilin province, Liaoning province, Heilongjiang province), North China (Beijing city, Tianjin city, Heber province, Shanxi Province (Taiyuan), Inner Mongolia), East China (Shanghai city, Anhui province, Jiangxi province, Jiangsu province, Zhejiang province, Fujian province, Shandong province,Taiwan), Central China (Henan province, Hubei province, Hunan province), South China (Guangdong province, Hainan province, Guangxi Zhuang Autonomous Region, Hongkong), Southwest of China (Chongqing City, Guizhou province, Sichuan Province, Yunnan Province, Tibet), Northwest of China (Shanxi province (Xi'an), Gansu province, Ningxia Hui Autonomous Region, Xinjiang Uyghur autonomous region).

M: model of meta-analysis; R: random-effects model; F: fixed-effects model.*P_H_: p* value of heterogeneity test. *P_E_*:*p v*alue of

Egger's test. *P_OR_*: *P*<0.001 replace the *P* = 0.000 and the *P* less than 0.001. @: *p* values could not be calculated.

NA: not available.

**Table 5:** Subgroup analysis of the association between *GSTM1-GSTT1* null genotype and lung cancer risk.

Polymorphism	Null vs. Present	No. of studies (cases/controls)	Odds ratio		M	Heterogeneity		*P_E_*
			OR [95%CI]	*POR*		*I^2^*(%)	*PH*	
*GSTM1-GSTT1*	All studies	8(775/1495)	1.29[1.03,1.63]	0.028	R	61.7	0.011	0.320
	subgroup analyses by number of case							
	<100	5(327/461)	1.33[1.07,1.65]	0.009	F	21.6	0.277	0.407
	≥100	3(448/1034)	1.30[0.84,2.00]	0.238	R	82.8	0.003	0.387
	subgroup analyses by source of control							
	Population-based	7(722/1423)	1.34[1.06,1.71]	0.016	R	64.5	0.010	0.126
	Hospital-based	1(53/72)	0.80[0.40,1.60]	0.528	R	@	@	@
	subgroup analyses by research design							
	Epidemiological study	7(698/1418)	1.34[1.03,1.73]	0.029	R	66.4	0.007	0.293
	Non-epidemiological study	1(77/77)	1.04[0.66,1.63]	0.864	R	@	@	@

M: model of meta-analysis; R: random-effects model; F: fixed-effects model.*P_H_*: *p* value of heterogeneity test. *P_E_*: *p* value of Egger's test. *P_OR_*: *P*<0.001 replace the *P* = 0.000 and the *P* less than 0.001. @: *p* values could not be calculated.

### 3. Cumulative meta-analysis

**Figure 5:**
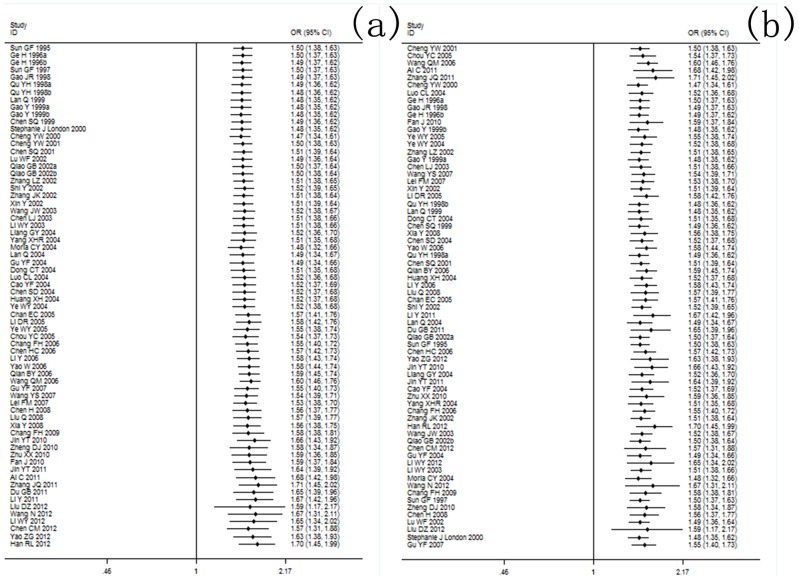
Cumulative meta-analysis of the association between GSTM1 null genotype and lung cancer susceptibility. (a) publication time cumulative meta-analysis of GSTM1 variants and lung cancer risk; (b) sample size cumulative meta-analysis of GSTM1 variants and lung cancer risk.

**Figure 6:**
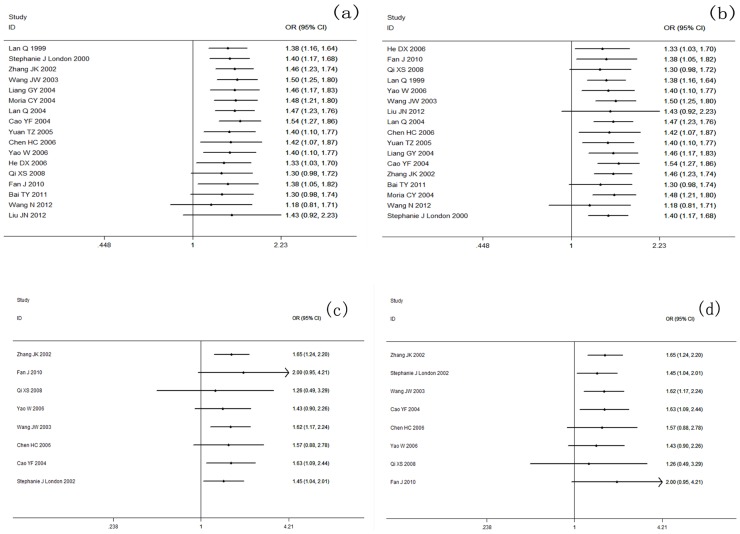
Cumulative meta-analysis of the association between *GSTT1*/*GSTM1-GSTT1* genetic polymorphisms and lung cancer susceptibility. (a) publication time cumulative meta-analysis of *GSTT1* variants and lung cancer risk; (b) sample size cumulative meta-analysis of *GSTT1* variants and lung cancer risk; (c) publication time cumulative meta-analysis of *GSTM1-GSTT1* variants and lung cancer risk; (d) sample size cumulative meta-analysis of *GSTM1-GSTT1* variants and lung cancer risk.

The cumulative meta-analysis was used to examine the fluctuation of the eligible studies with changes in the publication year or sample size. With the publication year development and sample size increase, the cumulative meta-analysis of *GSTM1* tended to be stable. However, no significant difference in the trend was found in the *GSTT1* and *GSTM1-GSTT1* cumulative meta-analysis. The results for cumulative meta-analysis are shown in Figure 5 and Figure 6.

### 4. Subgroup analysis

Due to the fact that all studies were middle to high heterogeneities, analyses on nine subgroups as mentioned above were performed accordingly. No significant increase in the risk of lung cancer was detected in either null genotype of *GSTM1* in the northwest, or null genotype of *GSTT1* in the north, southwest or northwest of China (Table 3, Table 4). The excess lung cancer risk was found associated with null *GSTM1* genotype, but not with null *GSTT1* genotype, in both smokers and nonsmokers. Besides, smokers had a higher risk than non-smokers in the association between *GSTM1* null genotype and lung cancer risk. The interaction of *CYP1A1* (Msp1) with mt/mt genotype and *GSTM1* null genotype could enhance the risk of lung cancer, and the OR of which were a little higher than the other two *CYP1A1* (Msp1) genotypes with *GSTM1* null.

However, high heterogeneities in the analysis of the association between *GSTM1* variants and lung cancer were found in the studies from northeast and southwest China. The subgroups of AC and smokers also showed greater heterogeneities (*I^2^*:53.8% and 50.3%, respectively). Meanwhile, the subgroup analyses of *GSTT1* genetic polymorphisms and lung cancer susceptibility demonstrated high heterogeneities in the subgroups of central China, southwest China, and smokers.

When analyzing the five subgroups of case numbers ≥100, population-based controls, epidemiological studies, test material from white blood cells, and quality score (7–8), all pooled results showed significant association between *GSTT1* genetic polymorphisms and lung cancer risk, but high heterogeneities also appeared. However, subgroups of case numbers <100, hospital-based controls, non-epidemiological studies, test materials from involved tissue or cells or not available, and quality score (4–5), all pooled results showed no significant association between *GSTT1* genetic polymorphisms and lung cancer risk (Table 4).

In the analysis of the relationship of *GSTM1-GSTT1* genetic polymorphisms with lung cancer risk, no significant association was found in the subgroup of case numbers (≥100). Along with significant increase risks in the subgroup of population-based controls and epidemiological studies, high heterogeneity was also found (Table 5).

### 5. Galbraith plot and sensitivity analysis

**Figure 7:**
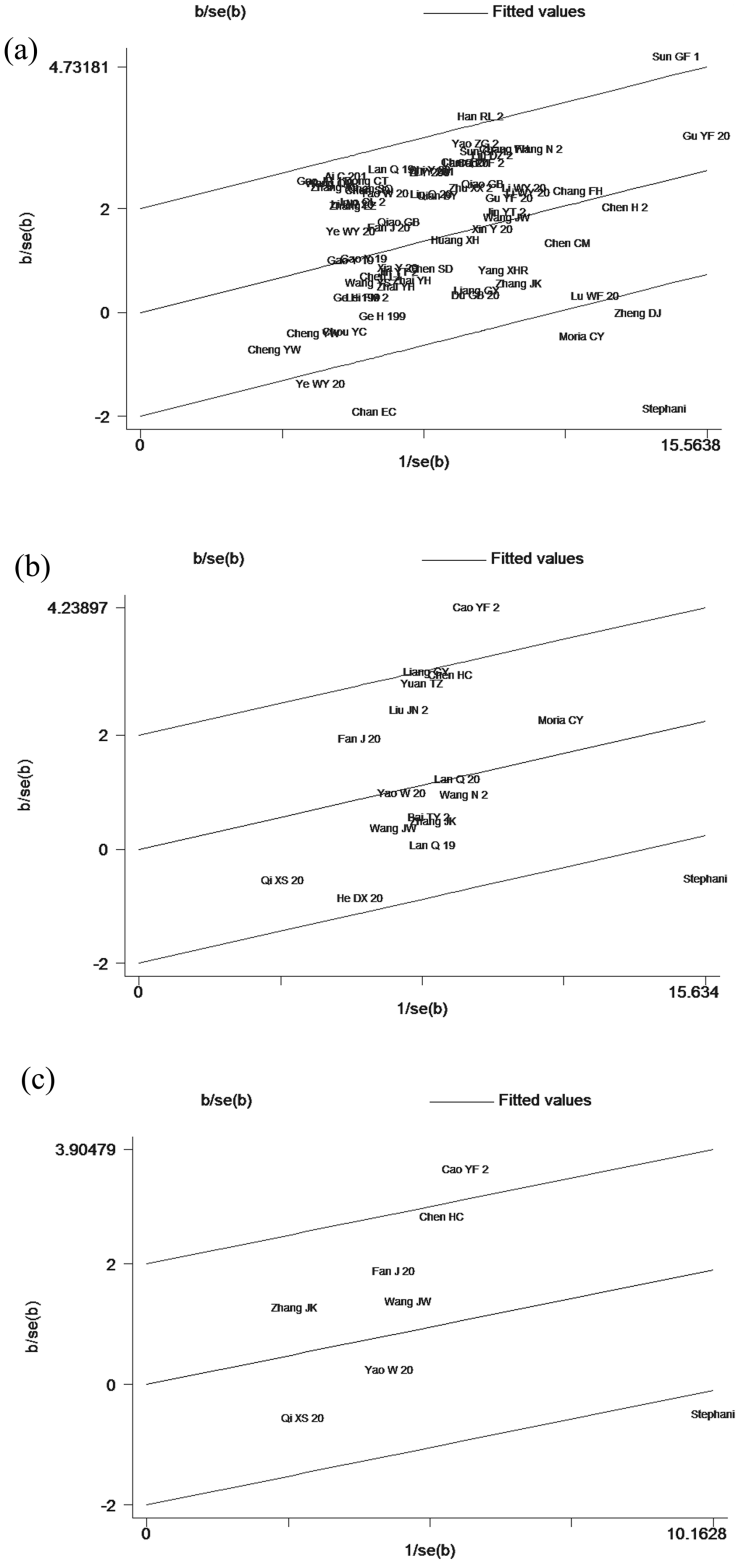
Galbraith plot of association between GSTs polymorphisms and lung cancer risk. Each figure represents a unique article in this meta-analysis. The figures outside the three lines were spotted as the outliers and the possible sources of heterogeneity in the analysis pooled from the total available number. (a) Galbraith plot result of GSTM1 polymorphisms and lung cancer risk; (b) Galbraith plot result of GSTT1 polymorphisms and lung cancer risk; (c) Galbraith plot result of GSTM1-GSTT1 dual null genotype and lung cancer risk.

In Figure 7a, 7 articles were identified in the Galbraith plot as the outliers [60], [68], [86], [89], [93], [115], [122]. After omitting these records, the adjusted association of *GSTM1* null genetype and lung cancer risk showed a lower heterogeneity and an increased susceptibility (fixed-effects model: OR = 1.23, 95% CI: 1.19 to 1.27, *P*<0.001). Besides, according to the Galbraith plot of the association of *GSTT1* or *GSTM1-GSTT1* interaction polymorphisms with lung cancer risk, 2 articles [98], [115] were obviously spotted as the outliers, which were the possible sources for the heterogeneities. After adjustment, the association of both groups were all increased (fixed-effects model: OR*_GSTT1_* = 1.18, 95% CI: 1.10 to 1.26, *P*<0.001; OR*_GSTM1-GSTT1_* = 1.33, 95% CI: 1.10 to 1.61, *P* = 0.004) and the *I^2^* indexes were decreased to 29.5% for *GSTT1* and 2.1% for *GSTM1-GSTT1*, respectively (Figure 7, Table 6). Then, the sensitivity analysis was carried out in each group (data not shown).

**Table 6:** Subgroup analysis of ^$^the adjusted association between *GSTM1* null genotype, *GSTT1* null genotype and *GSTM1-GSTT1* dual null genotype and lung cancer risk.

Polymorphism	Null vs. Present	No. of studies (cases/controls)	Odds ratio		M	Heterogeneity		*P_E_*
			OR[95%CI]	*POR*		I^2^ (%)	*PH*	
*GSTM1*	All studies	61(7455/8364)	1.23[1.19,1.27]	<0.001	F	2.2	0.427	0.337
*GSTT1*	All studies	15(1773/2116)	1.18[1.10,1.26]	<0.001	F	29.5	0.135	0.296
*GSTM1-GSTT1*	All studies	6(439/580)	1.33[1.10,1.61]	0.004	F	2.1	0.403	0.349

M: model of meta-analysis; R: random-effects model; F: fixed-effects model.PH: p value of heterogeneity test.PE: p value of Egger' test. POR: P<0.001 replace the P = 0.000 and the P less than 0.001. ^$^: adjusted association (after omitting several articles from Galbraith plot).

### 6. Potential publication bias

**Figure 8:**
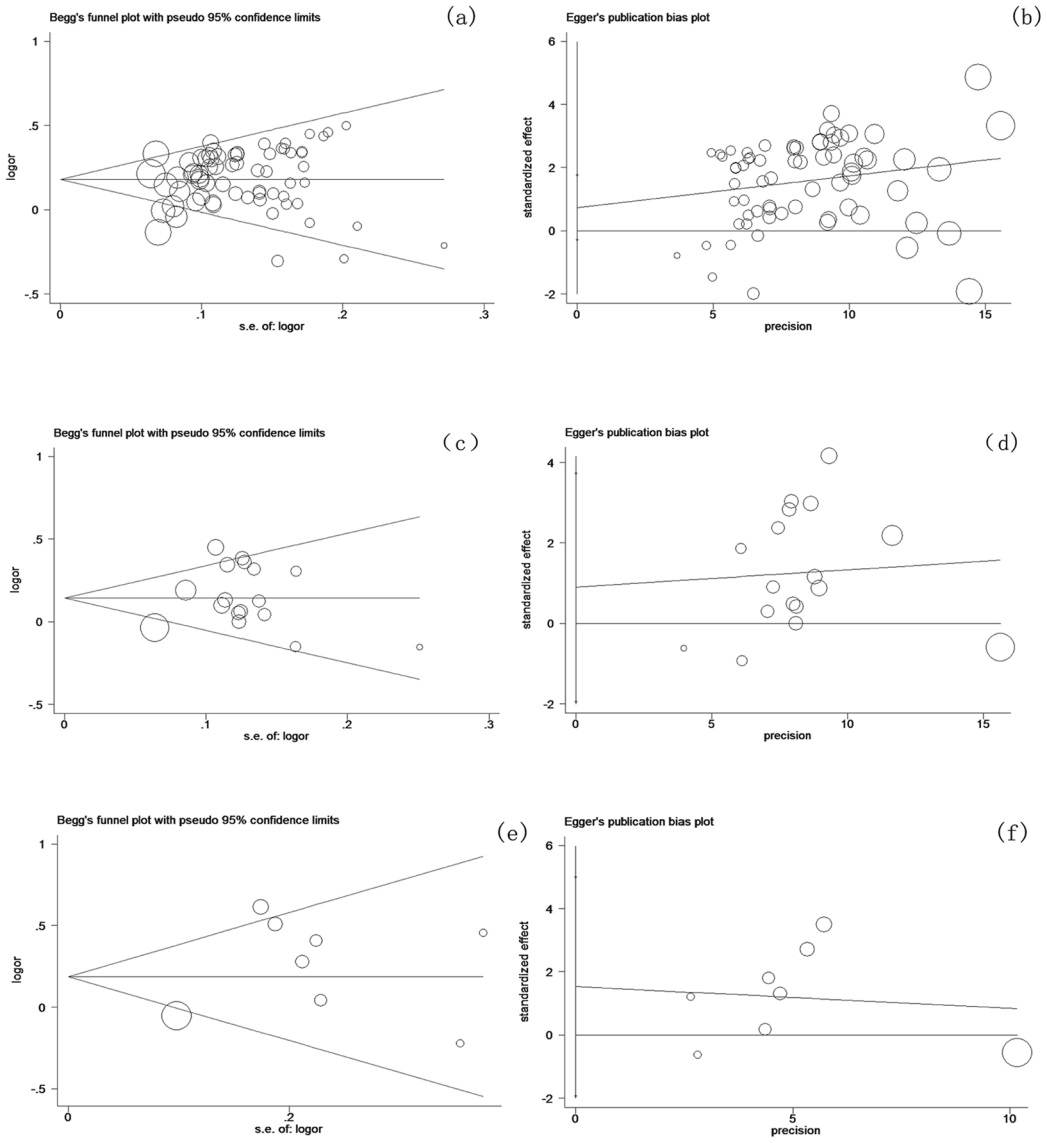
Begg's funnel plot and Egger's linear regression test of the association between *GSTs* polymorphisms and lung cancer risk. Begg's funnel plot is used to detect potential publication bias in which a symmetric funnel shape means no publication bias. Egger's linear regression test is used to quantify the potential presence of publication bias; (a) (b) *GSTM1*: No publication bias has been found from 68 inclusive studies about the association between *GSTM1* polymorphisms and lung cancer risk by Begg's??? test and Egger's test, respectively; (c)(d) *GSTT1*: No publication bias has been found from 17 inclusive studies about the association between *GSTT1* polymorphisms and lung cancer risk by Begg's test and Egger's test, respectively; (e)(f) *GSTM1-GSTT1* dual-null genotype: No publication bias has been found from 8 inclusive studies about the association between *GSTM1-GSTT1* dual-null genotype and lung cancer risk by Begg's test and Egger's test, respectively.

Begg's funnel plots and Egger's linear regression test were used to evaluate the potential publication bias (Figure 8a and Figure 8b for *GSTM1*; Figure 8c and Figure 8d for *GSTT1*; Figure 8e and Figure 8f for *GSTM1-GSTT1*). No publication bias was detected by Egger's test (*P_E_* = 0.245 for *GSTM1*, *P_E_* = 0.510 for *GSTT1* and *P_E_* = 0.320 for dual-null genotype of *GSTM1-GSTT1*). The Trim and Fill test further confirmed the results (data not shown).

## Discussion

To our knowledge, this is the first large-scale systematic meta-analysis on the correlation of two vital GSTs genetic polymorphisms with lung cancer risk in the Chinese population over the past decade. Our pooled analysis on the original studies in the Chinese population provided efficient and effective evidences of an increased association between null *GSTM1*, null *GSTT1* or dual null *GSTM1-GSTT1* genotypes and lung cancer risk when omitting some possible heterogeneous records. This large-scale systematic review on sufficient studies helps to reduce random error and increase the statistical power. Simultaneously, by using the same inclusive criteria, it can also ensure the pooled results more precise and exact. It is well known that different populations have different genetic variations and environmental exposure factors. Previous studies paid more attention to the Asian or special environmental population [35], [46]. We only focused on the Chinese ethnicity.

In subgroup analysis of *GSTM1* genetic variants, the northeast and southwest of China were found to be a source of difference, and in subgroup analysis of *GSTT1* genetic variants, the southwest regions of China was also suggested as the major heterogeneous source. Furthermore, no association between GSTs and lung cancer susceptibility was evident in the Chinese population living in the above regions. To our knowledge, the greatest population in the southwest and northwest areas of China is the Chinese ethnic minorities. The complex genetic backgrounds of various ethnic minorities might have an influence on lung cancer susceptibility. In the subgroup of histopathological classification, increased association between the genetic polymorphisms and SC (OR and 95% CI:1.20 [1.12,1.27]) and SCLC (OR and 95% CI:1.29[1.13,1.47]) risk were found with a low heterogeneity. These results for the first time imply a clue that SCLC could have a stronger association with *GSTM1* deficiency than the other two types while no statistic difference was found among 3 pathological types from available data. Due to the limited number of studies and comparatively diversity among various studies, more well designed epidemiological studies should be performed for various pathological types of lung cancers (especially for pulmonary AC). Additionally, we found that there was increased susceptibility between *GSTM1* null genotype and lung carcinoma risk in different phase I isoenzymes of *CYP1A1*. These results not only further confirm our conclusion, but also imply some enlightenments. For instance, under a higher OR with no heterogeneity, people with *CYP1A1* (mt/mt) and *GSTM1* null genotype should pay more attention to avoiding exposure to harmful environmental factors associated with lung cancer. Naturally, more studies including a genome-wide association study (GWAS) are necessary to prove this hypothesis. Due to the limited number of studies, the same analysis for the *GSTT1* null genotype was not performed.

The subgroup analyses of the smoking status for *GSTM1* studies further suggested that the possible risk factor of GSTM1 null genotype is different. However, eligible studies for G*STT1* failed to reach a significant association, which might be caused by a limited number of studies with high heterogeneities. Unclear smoking definition and inconsistent classification of the amount of tobacco consumed among different studies might all have an influence on the stability, reliability, as well as further in-depth analyses of the results. Therefore, clear smoking definition and consistent classification for the smoking status are necessary in any future research.

In the sensitivity analyses and Galbraith plot, 7 heterogeneous articles for *GSTM1* were detected by the Galbraith plot. The potential bias of these articles might be the result of small sample size, complex population composition, distinction of testing materials [86], and/or unknown reasons [115]. After omitting these articles, no heterogeneity was detected. Additionally, the Galbraith plot for the *GSTT1* and *GSTM1-GSTT1* groups spotted two of the same articles [98], [115] as the major source of between-heterogeneity. After removing these two articles, heterogeneity decreased substantially. Compared to the raw OR and 95% CI, the adjusted OR and 95% CI of *GSTT1* and *GSTM1-GSTT1* were both increased.

Cumulative meta-analysis showed a comparable change in the trend in the accumulated OR and 95% CI for *GSTT1* or *GSTM1-GSTT1* with the publication time development and sample size increase. Thus, to identify the real association between the *GSTT1* null type, *GSTM1-GSTT1* dual null type and lung cancer susceptibility, more large-scale case-control and cohort studies from multi-centers should be performed. At last, no publication biases were detected in our meta-analysis.

It's worth mentioning that Hardy-Weinberg equilibrium has been widely recommended in testing studies of genetic polymorphisms and diseases, the violations of which may have potential impacts on the results [125]. In this paper, no individual studies made any distinction between heterozygotes or homozygotes and *GSTM1* and *GSTT1* in the present genotype, so Hardy-Weinberg equilibrium tests could not be performed. Therefore, the Hardy-Weinberg equilibrium test results reported in some of the 71 articles might not be reliable.

It is worthy to note that several other limitations might be included in this study: (1) as common observational studies, case-control studies were susceptible to various biases (including recall bias of smoking status, different diagnostic criteria and the investigation bias of NOS score). These biases could influence the final findings of this study; (2) conclusions of this study were partly based on literatures obtained from the hospital-based population, which might not represent the whole population; (3) eligible studies for this study covered nearly all regions in China, but the article number was still insufficient in some less developed or relatively sparsely regions; (4) the interaction of genes with environmental factors, especially with special external occupational exposure and environmental pollution, might all contribute to the development of lung cancer. Factors above might also contribute to a possible source of heterogeneity of our results. Owning to the limitation of the data, this paper did not analyze the interaction effects of these factors; (5) absence of HWE test in the control group, some unbalance controls could lead to some bias in the final results.

Taken together, after a decade of extensive studying on this topic, our findings suggest that *GSTM1* and *GSTT1* genetic polymorphisms are associated with increased lung cancer risk in the Chinese population. Because of multifactor etiology of the interaction of gene-gene and gene-environment in the development of lung cancer, large-scale and methodologically sound studies with different environmental background and other genetic polymorphisms should be carried out to explore the real association between GSTs variants and various pathological types of lung cancer.

## Supporting Information

Checklist S1 **PRISMA checklist.**
(DOC)

Figure S1 **Map of the seven regions in China.**
(TIF)
