# Supplementary figures and images for: The Associations between Two Vital GSTs Genetic Polymorphisms and Lung Cancer Risk in the Chinese Population: Evidence from 71 Studies
Source: PLoS One. 2014 Jul 18;9(7):e102372. doi: 10.1371/journal.pone.0102372 (PMC4103841; doi:10.1371/journal.pone.0102372)

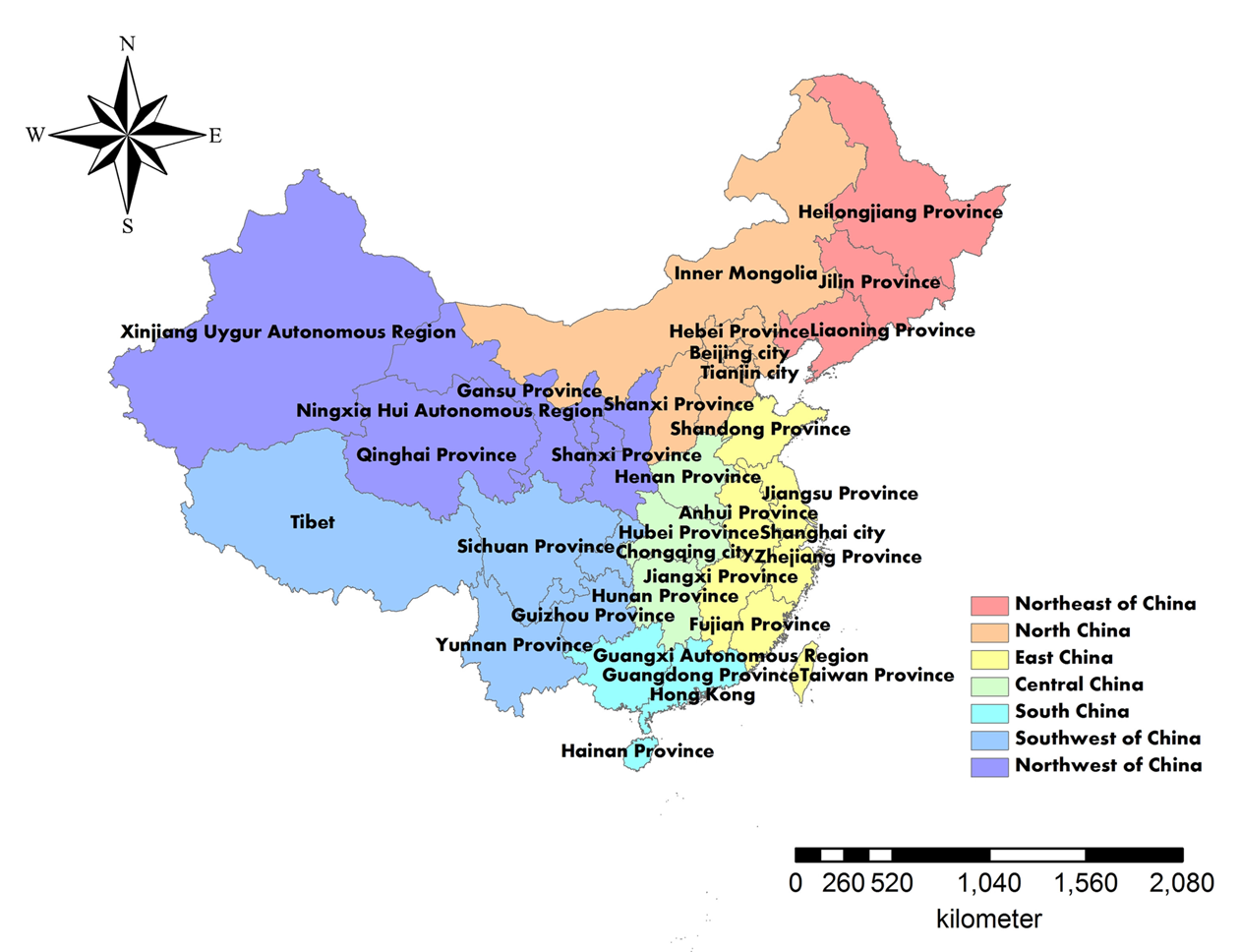

Supplement: Figure S1 — Map of the seven regions in China. (TIF) [file pone.0102372.s002.tif]
